# Efficacy of acupuncture in patients with mild Alzheimer’s disease and its impact on gut microbiota: Study protocol for a randomized sham-controlled trial

**DOI:** 10.3389/fmed.2023.1014113

**Published:** 2023-02-23

**Authors:** Xiehe Kong, Zhao Ma, Ran Tang, Xuejun Wang, Kai Wei, Guang Yang, Yanting Yang, Yue Zhao, Dan Zhang, Chen Xie, Gang Wang, Xiaopeng Ma

**Affiliations:** ^1^Yueyang Hospital of Integrated Traditional Chinese and Western Medicine, Shanghai University of Traditional Chinese Medicine, Shanghai, China; ^2^Shanghai Research Institute of Acupuncture and Meridian, Shanghai University of Traditional Chinese Medicine, Shanghai, China; ^3^Department of Neurology and Institute of Neurology, Ruijin Hospital, Shanghai Jiao Tong University School of Medicine, Shanghai, China; ^4^Shanghai Institute of Traditional Chinese Medicine for Mental Health, Shanghai Jiao Tong University School of Medicine, Shanghai, China

**Keywords:** acupuncture, Alzheimer’s disease, dementia, gut microbiota, study protocol

## Abstract

**Introduction:**

Acupuncture is used as an adjuvant therapy for Alzheimer’s disease (AD), but available evidence for efficacy is limited so far. Growing studies suggest that resident gut microbiota contributes to the development and progression of AD. Meanwhile, acupuncture is reported to treat gastrointestinal and neurodegenerative disorders *via* the gut-brain axis. Therefore, our aim is to confirm the adjunctive therapeutic effects of acupuncture for AD, and explore the relationship between clinical efficacy and shifts of gut microbiota.

**Methods and analysis:**

This is a randomized, participant-masked, sham-controlled trial. One hundred and sixty participants with mild AD will be randomly assigned (1:1) to either active acupuncture or non-penetrating sham acupuncture (three times weekly for 14 weeks) added to donepezil treatment (5 mg per day for 28 weeks). The primary efficacy outcome is the change from baseline to week 28 in the Alzheimer’s disease Assessment Scale (ADAS-cog12). Secondary efficacy outcomes include other assessments of the Mini-Mental State Examination (MMSE), the Alzheimer’s disease Cooperative Study-Activities of Daily Living (ADCS-ADL), and Neuropsychiatric Inventory (NPI). Gut microbiota will be measured using 16S rRNA tag sequencing.

**Discussion:**

This rigorous trial will provide high-quality evidence on the efficacy of acupuncture as adjunctive treatment for mild AD, and identify the possible mechanisms of acupuncture from gut microbiota.

**Clinical Trial Registration:**

[https://clinicaltrials.gov/ct2/show/NCT05078944], ClinicalTrials.gov [NCT05078944]. Registered 15 October 2021

## Introduction

In 2019, Alzheimer’s Disease International estimated that there were over 50 million people living with dementia globally, a figure set to increase to 152 million by 2050 (1). Alzheimer’s disease (AD) is the most common type of dementia in older people. There have been more than 100 attempts to develop an effective drug for AD since 1998, but only four drugs have been authorized and widely used (2–5). The currently available drug treatments such as donepezil and memantine are aimed at cognitive enhancement and typically work through neurotransmitter mechanisms, they only improve symptoms of AD rather than slow down the progression of the disease (6). Thus, new or adjuvant therapies are urgently needed.

Acupuncture is one of the most commonly promoted complementary and alternative therapies. Emerging evidence from both animal (7) and human studies (8–10) supports acupuncture as a candidate anti-AD therapy. However, current trials provided insufficient evidence to determine the effectiveness of acupuncture to treat AD because of methodological flaws. To the best of our knowledge, only one study (11) used single-blinded design to test the specific effect of acupuncture by the comparison of acupuncture plus donepezil placebo versus sham acupuncture plus donepezil, but no significant between-group difference was found in primary parameters of cognitive function. Due to the absence of a control group with sham acupuncture plus donepezil placebo, the question as to whether acupuncture has a specific treatment effect rather than a placebo effect for AD remained unconfirmed. A recent meta-analysis (10) indicated that acupuncture plus medications may have a more beneficial effect for AD patients than drug therapy alone on general cognitive function, while acupuncture alone may not have superior effects compared with drug therapy. From this, the focus on examining the additive effect of acupuncture in combination with standard care for AD seems more valuable in a pragmatic context.

The high failure rates of β-amyloid (Aβ)- or tau-centric therapeutic strategies in late-stage clinical trials indicate the current understanding of the pathogenesis of AD must be extended (12, 13). While it is known that Aβ plaques and intracellular neurofibrillary tangles contribute heavily to neuronal death, it is unclear as to why these proteins accumulate in the first place. In recent years, a plethora of studies have indicated that the development of AD can be linked to gut microbiota that naturally reside in the body based on the following results: (1) Microbiome profiling of both AD patients (14, 15) and mouse models (16, 17) for AD has suggested alterations in the gut microbiota; (2) Modulations of the gut microbiota through germ-free rearing (18), dietary alterations (19), antibiotic treatment (20) or fecal microbiota transplantation (21, 22) alter learning and memory-related behaviors. Further, acupuncture was reported to treat diseases such as abdominal obesity (23) and irritable bowel syndrome (24, 25) by gut-brain axis. A recent study (26) demonstrated that the acupuncture at Baihui (GV20), Yintang (GV29), and Zusanli (ST36) benignly modulated gut microbiota dysbiosis and improved cognitive function in AD mouse model. Collectively, it is suggestive that gut microbiome-brain axis may be a potential mechanism of acupuncture for AD.

This trial intends to reveal if adjunctive acupuncture will slow down the cognitive decline as compared with sham acupuncture in participants with mild AD, as well as the relationship between clinical efficacy and gut microbiota.

## Methods and analysis

### Study design

This is a prospective, randomized, participant-masked, sham-controlled trial, with each eligible participant randomly assigned to either active or sham acupuncture added to donepezil treatment. All participants or their legal representatives will provide written informed consent and be treated in accordance with the tenets of the Declaration of Helsinki (version 2013).

### Participant recruitment

**Figure 1:**
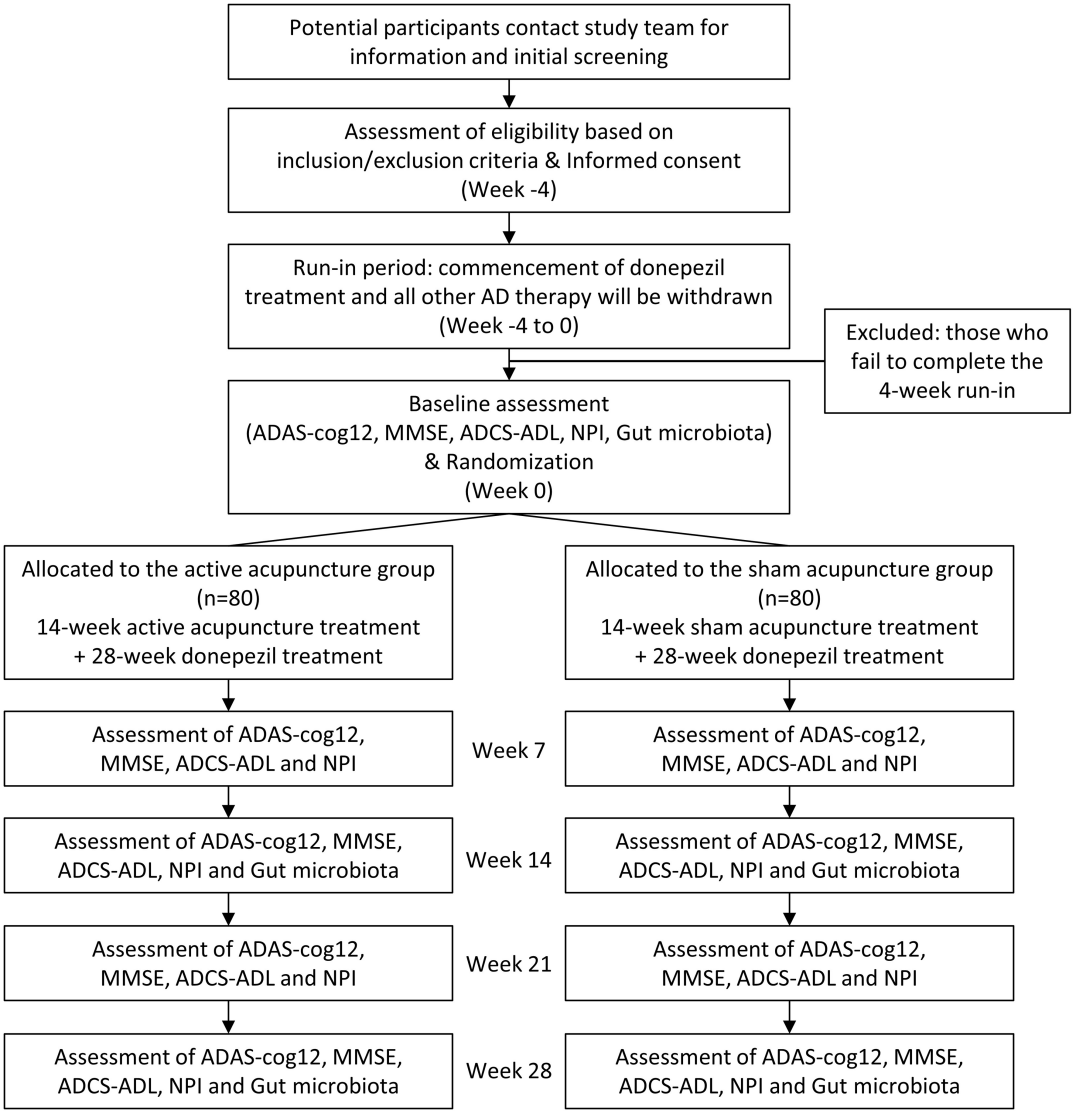
Flow diagram of the study protocol. ADAS-cog12, Alzheimer’s Disease Assessment Scale; MMSE, Mini-Mental State Examination; ADCS-ADL, the Alzheimer’s Disease Cooperative Study Activities of Daily Living; NPI, Neuropsychiatric Inventory.

The study will take place in the Outpatient Department, Shanghai Research Institute of Acupuncture and Meridian, Shanghai, China, and the recruitment will be announced *via* our official account on social media. Placement of brochures and study posters in Yueyang Hospital of Integrated Traditional Chinese and Western Medicine, Shanghai University of Traditional Chinese Medicine and Ruijin Hospital, Shanghai Jiao Tong University School of Medicine (collaborating hospitals), and community centers will be used to assist recruitment. Participants or their representatives will be instructed to read the informed consent and those who showed interest in the study will be scheduled for the screening visit. After presenting written informed consent, participants will be examined, and if all entry criteria are met, will commence a 4-week run-in period of treatment with donepezil hydrochloride (5 mg/tablet, China) 5 mg daily (prior AD treatments will be terminated by then). To further avoid potential confounding effects on the clinical outcomes and gut microbiota, participants will be instructed to maintain their regular diet and activity level, and refrain from probiotics, prebiotics, or synbiotics use. Upon successful completion of the 4-week run-in, participants will be scheduled to complete baseline assessments and undergo randomization. The study flowchart and proposed trial schedule are shown in Figure 1 and Table 1, respectively.

**Table 1:** Study schedule.

Visit No.	V1	V2	V3	V4	V5	V6
End of week relative to study treatment start	−4	0	7	14	21	28
Tolerance interval for visit (days)	0	0	±3	±3	±7	±7
Entry and administrative						
Eligibility	X	X				
Informed consent	X					
Physical/neurological examination	X					X
Demographics	X					
Medical history	X					
Randomization		X				
Interventions						
Active/Sham acupuncture		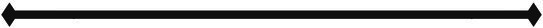				
Donepezil	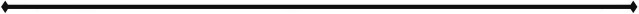					
Outcomes measures						
ADAS-cog12		X	X	X	X	X
MMSE		X	X	X	X	X
ADCS-ADL		X	X	X	X	X
NPI		X	X	X	X	X
Fecal samples collection		X		X		X
Additional assessment						
Adverse events		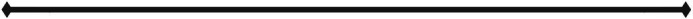				
Treatment compliance		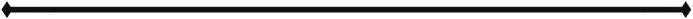				

ADAS-cog12, Alzheimer’s Disease Assessment Scale; MMSE, Mini-Mental State Examination; ADCS-ADL, the Alzheimer’s Disease Cooperative Study Activities of Daily Living; NPI, Neuropsychiatric Inventory.

### Inclusion criteria

- Aged 50–80 years (inclusive), no gender limitation.
- Meets the diagnostic criteria for AD (27).

Diagnostic criteria include:

- dementia established by clinical examination and documented by the Mini-Mental State Examination, Blessed Dementia Scale (MMSE), or some similar examination, and confirmed by neuropsychological tests.
- deficits in 2 or more areas of cognition.
- progressive worsening of memory and other cognitive functions.
- no disturbance of consciousness.
- onset between ages 40 and 90, most often after age 65.
- absence of systemic disorders or other brain diseases that, in and of themselves, could account for the progressive deficits in memory and cognition.

Supported by:

- progressive deterioration of specific cognitive functions such as language (aphasia), motor skills (apraxia), and perception (agnosia).
- impaired activities of daily living and altered patterns of behavior.
- family history of similar disorders, particularly if confirmed neuropathologically.
- laboratory results of normal lumbar puncture as evaluated by standard techniques, normal pattern or nonspecific changes in electroencephalogram, and evidence of cerebral atrophy on CT with progression documented by serial observation.

- Scored 1.0 by the Clinical Dementia Rating Scale (CDR) Global Score.

### Exclusion criteria

- Evidence of a clinically relevant or unstable psychiatric disorder.
- Has unstable or severe cardiovascular, hepatic, renal, respiratory, endocrinologic, neurologic diseases, and other conditions that, in the investigator’s opinion, could interfere with the analyses of safety and efficacy in this study.
- Use of AD therapy (except for donepezil hydrochloride) which cannot be stopped.
- Has visual or hearing disorder, defeating completion of evaluation.
- Without a reliable caregiver who will accompany the participant during treatment and assessment, and monitor administration of the prescribed medications.
- Has irritable bowel syndrome or inflammatory bowel disease.
- Use of antibiotics within 1 month prior to enrollment.
- Has a history of gastrointestinal surgery (except for appendicitis and hernia surgery).
- With cardiac pacemaker or metal allergy.
- Once experienced electroacupuncture treatment before at any time (manual acupuncture is allowed).
- Premenopausal woman.

### Randomization and blinding

Eligible participants are randomly allocated to active or sham acupuncture group at a ratio of 1:1 *via* a computer-generated randomization. The randomization sequence is generated by a third party in permuted blocks of randomly varying sizes using the “proc plan” procedure of the SAS 9.4 (SAS Institute Inc.). Consecutively numbered opaque sealed envelopes for each group are stored in a secure location and opened sequentially upon enrollment of a study participant by the research assistant. The participants, assessors and the statisticians, except the licensed acupuncturist, are masked to the treatment allocation. Participants will be informed about the active and sham acupuncture in the study as follows (28): “In the present study, different types of acupuncture will be compared. One acupuncture protocol is based on previous studies. The other acupuncture protocol is chosen as a contrast and has also been associated with positive outcomes in clinical studies. There is no evidence that either acupuncture protocol being utilized is more effective than the other, which is why we are conducting this research.” To avoid communication, the participants will be treated and assessed separately at different times during the trial. Moreover, the credibility questionnaire will be used after the 1st and the final acupuncture sessions as a check that the active and sham treatments are equivalent in their psychological impact. The treatment allocation will only be unblinded if there is a serious medical or safety reason.

### Interventions

**Figure 2:**
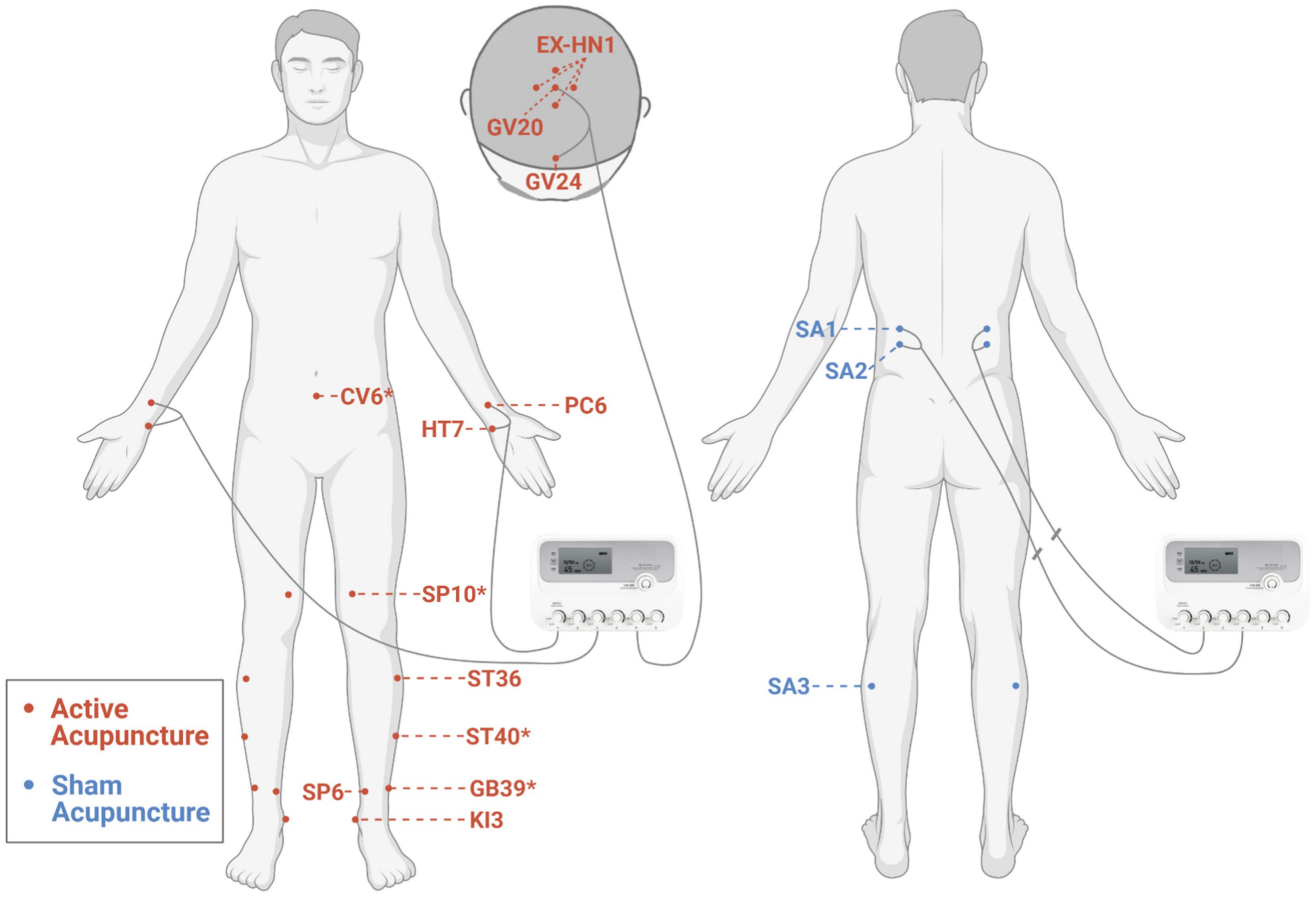
Locations of acupoints for the active and sham acupuncture. *Additional acupoints. The figure was created with BioRender.com.

Acupuncture will be performed by an experienced, licensed acupuncturist. Acupuncture intervention is established under guidelines of Revised Standards for Reporting Interventions in Clinical Trials of Acupuncture (STRICTA) (28). A summary of acupuncture points, locations, and insertion details is presented in Figure 2 and Supplementary Table 1.

According to previous Delphi expert consensus survey (29) and our data mining study (30), participants in the active acupuncture group will receive acupuncture at Baihui (GV20), Sishencong (EX-HN1), Shenting (GV24), bilateral Neiguan (PC6), Shenmen (HT7), Zusanli (ST36), Taixi (KI3), and Sanyinjiao (SP6). Additional acupoints are selected on the basis of traditional Chinese medicine (TCM) syndrome differentiation: bilateral Xuanzhong (GB39) for syndrome of brain marrow deficiency, Qihai (CV6) for syndrome of dual deficiency of Qi and blood, bilateral Fenglong (ST40) for syndrome of orifices blocked by phlegm, and bilateral Xuehai (SP10) for syndrome of blood stasis. Sterile stainless steel disposable acupuncture needles (size 0.30 mm × 50 mm, Hwato brand, China) are used. After sterilization, a small plastic ring (diameter 10 mm and height 5 mm) will be fixed over the acupuncture point (except for the acupoints on the head) with plaster to facilitate maintenance of blinding for the participant, and then, the needle will be inserted through the plaster ring. Acupuncturists will then try to elicit a dull and aching sensation by lifting, thrusting, and/or rotating manipulation of needles as well as perceive a “bait-biting” sensation caused by mechanical interaction between the needle and connective tissue (31). These sensations are indicative of proper needle placement (traditionally termed “De Qi”). The needle handles at GV20, GV24 (32), bilateral PC6, and HT7 (33, 34) will be connected to an electroacupuncture apparatus (SDZ-III electroacupuncture apparatus, Hwato brand, China) and stimulated with dilatational wave, 10/50 Hz, and tolerable electric current. Needles placed in other acupoints will be manually stimulated by rotation every 15 min. Each treatment session lasts for 45 min and is conducted in a quiet treatment room with dimmed light. Acupuncture treatment in this study is to be taken three times weekly over a period of 14 weeks (42 sessions).

For the sham acupuncture group, non-penetrating sham acupuncture will be performed at bilateral sham acupoints (SA1, SA2, and SA3). After sterilization, same plastic rings are placed at the sham acupoints and pragmatic placebo needles with blunt tip (size 0.30 mm × 50 mm, Hwato brand, China) are used (35). When the needle tips are pressed against the skin through plastic rings, participants will feel a pricking sensation. The electric stimulator will be applied to bilateral SA1 and SA2 with no current output. The internal electrical wires are disconnected (see Supplementary Image 1) with a same outlook as the electroacupuncture group. The wave type, frequency, and time are presented on the display screen of the device during each treatment, indicating a “running” status. Needles placed in SA3 will be manually rotated every 15 min. To best simulate contextual aspects of acupuncture, sham needles are taken out from their packaging right before insertion and cast away in a sharps container after treatment. The number, duration, and frequency of sessions in the sham acupuncture group are the same as in the active acupuncture group.

In both groups, we consider participants who attend 85% or more (≥36 of 42) of acupuncture sessions and take at least 12 times of donepezil hydrochloride in every 14 consecutive days to have completed a full course of treatment. Concomitant donepezil treatment will be continued unchanged during the study. Participants will return the unused tablets at each follow-up visit. Number of acupuncture sessions and unused tablets will be counted and recorded. Any other concomitant treatments for AD are considered to violate the study protocol, for which participants will be withdrawn.

### Study assessments

According to a systematic review (36) which identified 81 outcome measures used across trials in mild-to-moderate dementia, recommended core outcomes for future disease modification trials were cognition as the fundamental deficit in dementia, and cognition should be measured by Alzheimer’s Disease Assessment Scale-Cognitive Subscale (ADAS-cog) or MMSE. Considering that the ADAS-cog is more sensitive to change than the MMSE, we set the primary efficacy measure in the present study as the change from baseline to 28 weeks in scores on the 12-item cognitive subscale of the ADAS-cog (37). The ADAS is a rater-administered instrument that was designed to assess the severity of dysfunction in the cognitive and noncognitive behavior characteristics of persons with AD. The cognitive subscale of the ADAS consists of 11 items assessing areas of cognitive function most typically impaired in AD: orientation, verbal memory, attention, reasoning, language, and praxis. The 12th item “concentration/distractibility” is added. The ADAS-cog12 scale ranges from 0 to 75, with higher scores indicating greater cognitive impairment.

Secondary efficacy measures include changes in the scores of the following assessments: the MMSE (score ranges from 0 to 30; a lower score indicates greater disease severity) (38); the Alzheimer’s Disease Cooperative Study-Activities of Daily Living (ADCS-ADL; score ranges from 0 to 78, with lower scores indicating greater functional impairment) (39); and Neuropsychiatric Inventory (NPI; score ranges from 0 to 144, with lower scores indicating fewer behavioral disturbances) (40).

16S rRNA gene sequencing analysis will be performed. Bacterial diversity will be determined by α-diversity (Chao1 richness estimator, Abundance-based Coverage Estimator metric, Shannon diversity index, and Simpson index) and β-diversity (Principal coordinates analysis, PCoA). To further reveal the linking hypotheses of “acupuncture-gut microbiota-AD” axis, the functional profile of Kyoto Encyclopedia of Genes and Genomes (KEGG) Orthology for each sample will be predicted. The participants and their caregivers will be instructed under tutoring video to collect a fresh fecal sample using sample collection kits, packaged within insulated containers and chilled with frozen gel packs. All fecal samples will be dispensed in 2 ml Eppendorf tubes and immediately stored in −80°C until analysis. Total bacterial genomic DNA samples are extracted using the Fast DNA SPIN extraction kits (MP Biomedicals, Santa Ana, CA, United States), following the manufacturer’s instruction. DNA quantity is determined using the NanoDrop1000 spectrophotometer (Thermo Fisher Scientific, Waltham, MA, United States), and quality is assessed by 1.0% agarose gel electrophoresis. PCR amplification of the bacterial 16S rRNA gene V3–V4 region is performed in a multiplex approach. The amplicons will be purified using Agencourt AMPure XP Beads (Beckman Coulter, Indianapolis, IN, United States) and quantified using the PicoGreen dsDNA Assay Kit (Invitrogen, Carlsbad, CA, United States). Then, the final equimolar pool will be sequenced using an Illumina MiSeq platform according to the manufacturer’s recommendations. The Quantitative Insights into Microbial Ecology (QIIME, v1.8.0) pipeline is employed to process the sequencing data (41). Operational taxonomic units (OTUs) are defined by clustering sequences using the UCLUST algorithm and a similarity threshold of 97% against the Greengenes database.

The treatment credibility scale will be assessed at the end of session 1 and session 42 by asking participants to rate their response to four questions on a 5-point scale developed by Borkovec and Nau (42). The first assessment allows us to test the participants’ blinding before sustained efficacy is evident. The second assessment will allow us to detect the association of efficacy with beliefs about treatment received.

The safety of the acupuncture intervention is evaluated by documenting any symptoms possibly related to acupuncture at each acupuncture visit, which includes subcutaneous hematoma, local errhysis at acupoints, sharp pain, palpitation, nausea, dizziness, and faint during acupuncture. All adverse events will be carefully documented throughout the study period. For each Data and Safety Monitoring Board (DSMB) report, a list and summary of the reported adverse events will be presented in a blinded fashion, unless otherwise formally requested.

After the 14-week acupuncture intervention, monthly phone calls will be made to maximize retention and loss to follow-up. Participants may withdraw from the study for any reason at any time. Their assessment data up to the timepoint that they withdrew will be used and such patients will not be replaced.

### Date management

An electronic data capture system will be used in this study for data entry management. When the data entry is complete, the database will perform consistency check automatically. On trial-specific documents, except for the signed consent, the participant will be referred to by a unique trial-specific code in any database, not by name. All data sets will be password protected. Modifications to data stored in the electronic database will be documented *via* a data change system. All paper documents are to be stored in numerical order and maintained safely in confidential conditions for a period of 3 years after completion of the study. The project investigator will have access to all data sets.

### Sample size calculation

Sample size was estimated using SAS version 9.4, with ADAS-cog12 as the designated primary outcome measure. Based on the results of previous studies (10), we assume a treatment difference of approximately 3.0 with a standard deviation of 6.0. Considering a 10% non-adherence to treatment and a 10% loss to follow-up, 80 randomized patients per arm or 160 randomized patients in total (128 completers), will have more than 80% power and a type I error rate of 5% for the ADAS-cog12 comparison to detect a significant treatment difference.

### Statistical analysis

Analyses will be conducted on the basis of a modified intention-to-treat principle and involve only patients who have outcome measurements both at and after baseline. The missing data will be imputed using last observation carried forward method. Supplementary per-protocol analysis will also be carried out. Continuous data are tested for normality first. Baseline characteristics between the two treatment groups will be evaluated by unpaired *t*-test or Mann–Whitney *U* test for continuous data and Chi-square test or Fisher’s exact test for categorical data. The primary outcome, the change from baseline in the ADAS-cog12 score, will be analyzed using a mixed-model repeated-measures analysis, with the change from baseline at each scheduled visit at weeks 7, 14, 21, and 28 as the dependent variables. The model for the fixed effects includes gender, TCM syndrome, education level, treatment, visit, and treatment-by-visit interaction, with age and the baseline value of the dependent variable as continuous covariates. Least squares means and 95% confidence interval will be estimated to compare outcomes between the active and sham acupuncture groups. The same approach is used for the change from baseline in the MMSE, ADCS-ADL, and NPI scores. Ancillary analyses will also be undertaken to confirm the treatment effects across several subgroups (TCM syndrome, age, gender, and education level). For any variables, which show an abnormal distribution, non-parametric Wilcoxon test will be performed. All the tests of effects are conducted at a two-sided alpha level of 0.05 using SAS version 9.4.

16S rRNA gene sequencing analysis is performed using QIIME and R packages (v3.2.0). OTU-level alpha diversity indices are calculated using the OTU table in QIIME. OTU-level ranked abundance curves are calculated to assess OTUs richness and evenness among samples. Beta diversity analysis will be performed to investigate the structural variation of microbial communities across samples using UniFrac distance metrics and visualized *via* PCoA. The linear discriminant analysis (LDA) effect size method is used to characterize the taxa with statistical significance and biological relevance. The set value of the LDA score is 2. Partial least-squares-latent structure discriminate analysis is performed using Simca-P 14.0 (Umetrics AB, Sweden) to observe the fecal microbiota pattern in different groups based on OTUs of the sequencing data from each sample. Variables with variable importance in projection >1 are identified as important contributors to generation of the model. The functional profile of KEGG Orthology for each sample will be predicted from 16S rRNA amplicon data with Phylogenetic Investigation of Communities by Reconstruction of Unobserved States (43). To test the overall correlation between changes in microbiome and altered outcome variables, pairwise Spearman’s rank correlations within the active and sham acupuncture groups at baseline and 14 and 28 weeks are calculated and adjustments are performed using the Benjamini–Hochberg procedure and Spearman’s rho values are filtered with a false discovery rate at 5%.

### Trial monitoring

An independent DSMB is established to review accumulating study data on a periodic basis and make recommendations to protect the safety of the participants. The members of the DSMB include a recognized expert in the field of AD, a senior acupuncturist, and a statistician (Supplementary Table 2). All members are external to our institute. Only the DSMB is authorized to evaluate unblinded interim efficacy and safety analyses. An interim analysis is proposed to be conducted at stage of 1/2 enrollment.

### Trial status

The trial was commenced on June 2022 and is currently at the stage of recruiting patients.

## Discussion

The present trial will provide convincing evidence to confirm that acupuncture has a therapeutic effect, rather than a placebo effect as adjunctive treatment for mild AD. Meanwhile, we also will explore the relationship between clinical efficacy and shifts of gut microbiota at first time.

Blinding is a challenge in trials of nonpharmacologic modality. To date, the commonly used sham controls in acupuncture studies can be divided into two categories: superficial needle insertion at sham acupoint and non-penetrating stimulation to real/sham acupoint with blunt needle. A systematic review argued that either the needle is inserted into a real acupoint or a sham acupoint could produce a physiological effect (44). Further, evidence showed that a light touch can provide effective stimulus for C tactile afferents and thus play a role in reducing pain (45), so a stronger stimulus, such as needle penetration stimulation, may result in greater neurological responses related to the treatment effect and should be avoided in clinical trials. As the receptive field of the disease-related acupoints could be expanded during illness according to the theory of “acupoint sensitization” (46), we set the sham acupoints far away from the active acupoints as opposed to 1–2 cm besides the active acupoints as used by most of the sham-controlled acupuncture studies. Collectively, our non-penetrating sham acupuncture protocol minimizes the physiological effects of the needling while maintaining its psychological impact. Streitberger (47) and Park sham device (48) have been validated most in acupuncture studies. However, the appearance and complicated manipulation of these sham devices differ significantly from conventional acupuncture device. For Chinese participants, who are relatively familiar with acupuncture procedures compared with the western participants, these dissimilarities may lead to mistrust or even dropout. To tackle this problem, a validated sham acupuncture device which targets Chinese population will be used in our trial (35). Moreover, besides simulation of needling procedure, contextual cues will also be performed to further enhance blinding through removing needles from package and simulating sounds associated with sharps disposal.

We previously performed a data mining analysis by reviewing the clinical literatures on acupuncture for AD published during the last decade (30). The principle acupoints selected in our acupuncture protocol are among the top 10 acupoints most frequently used for AD, which ensures the effectiveness to a certain degree. The effects of acupuncture can be further enhanced by electrical stimulation or manual manipulation. Electroacupuncture is believed to be more repeatable, and more effective in ameliorating the spatial learning and memory capability (49). The parameters of electroacupuncture, including waveform and frequency, are also important factors influencing treatment response. With the evidence available in the present time, dilatational wave seems more effective in improving the cognitive function when compared with continuous wave (either low or high frequency) and intermittent wave (50–52). Hence, we consider 10 Hz/50 Hz dilatational wave as the optimal waveform. A Delphi expert consensus survey (29) was conducted last year to guide acupuncturists in implementing clinical acupuncture practice for cognitive impairment. The results suggested that syndrome differentiation treatment, longer needle retention time (30–60 min per session), high frequency of treatment (at least three sessions per week), and 3 months of continuous treatment contribute to the favorable effectiveness of acupuncture for cognitive impairment. We followed all the items mentioned above in our design to provide compelling evidence for the efficacy of acupuncture for AD. Donepezil, a first-line AD medication approved by FDA, is selected as the basic intervention, ensuring that participants can receive the standard care for AD. A 4-week run-in period of treatment with donepezil is designed to reduce its confounding effects on clinical outcomes and gut microbiome. Besides, the potential dropouts due to adverse effects with donepezil can partly be controlled.

A wealth of studies indicated that the therapeutic effects of acupuncture on AD can be exerted *via* multiple targets and pathways (7), which showed a reasonable agreement with this therapy’s characteristics of systemic regulation. Nevertheless, the bulk of these studies only focused on single downstream targets, such as Aβ or tau, with less consideration on the mechanism from a holistic view. With repeated failures of single-target therapeutic treatments in the last decade, the intimate relationships between microbial dysbiosis and several hallmark features of AD have drawn increasing attention. Therefore, from a systems biology perspective, it is preferred to study the mechanisms of acupuncture for AD underlying gut microbiota.

Our study protocol has some limitations. First, it should be noted that we do not adopt a multicenter design as investigators are difficult to be continuously trained to ensure consistency in performing acupuncture and assessing clinical data. However, we believe that this rigorous trial will provide high-quality evidence for the efficacy of acupuncture as adjunctive treatment for mild AD, and explore the mechanisms of acupuncture from gut microbiota. Second, this protocol cannot answer whether the therapeutic effect of acupuncture on AD is dependent on specific acupoints. Adding a group with acupuncture at non-AD-related acupoints in future studies may provide an answer.

## Ethics and dissemination

The protocol (version 2, 10 May 2021) and informed consent were approved by the Medical Ethics Committee of Yueyang Hospital of Integrated Traditional Chinese and Western Medicine, Shanghai University of Traditional Chinese Medicine (No. 2021-052). The chief investigator will submit and obtain approval from the above party for all substantial amendments to the original approved documents. Written informed consent will be obtained from all eligible participants or their legal representatives. No later than 2 years after the completion of the trial, we will deliver a completely deidentified data set to an appropriate data archive for sharing purposes.

Acupuncture treatments and all assessments are provided free of charge, while the donepezil hydrochloride tablets are not free. Participants will also receive financial compensation for transportation.

The group will make every effort to prevent possible harms due to this study. If an adverse event occurs during a clinical trial, a medical expert committee will determine whether it is related to the trial. The participants who suffer from trial participation will be given economic compensation.

The investigators will be involved in reviewing the manuscripts, abstracts, press releases, and any other publications arising from the study. Authorship will be determined in accordance with the ICMJE guidelines and other contributors will be acknowledged.

## Ethics statement

The studies involving human participants were reviewed and approved by Medical Ethics Committee of Yueyang Hospital of Integrated Traditional Chinese and Western Medicine, Shanghai University of Traditional Chinese Medicine, China. The patients/participants provided their written informed consent to participate in this study.

## Author contributions

XM and XK conceived the study. ZM, XW, GY, and YY designed the study. KW and RT performed the sample size calculation and provided statistical analysis plan. XW, YZ, and DZ contributed to the methods of sham acupuncture. GW and CX contributed to the definition of primary and secondary outcomes. XK and ZM wrote the original draft. XW, RT, and XM revised the manuscript. All authors contributed to the article and approved the submitted version.

## Funding

XK, GW and XM obtained funding from Shanghai Municipal Health Commission (20204Y0251, ZHYY-ZXYJHZX-202006) and Outstanding Leader Plan of Shanghai (No. 060).

## Conflict of interest

The authors declare that the research was conducted in the absence of any commercial or financial relationships that could be construed as a potential conflict of interest.

## Publisher’s note

All claims expressed in this article are solely those of the authors and do not necessarily represent those of their affiliated organizations, or those of the publisher, the editors and the reviewers. Any product that may be evaluated in this article, or claim that may be made by its manufacturer, is not guaranteed or endorsed by the publisher.

## Supplementary material

SUPPLEMENTARY FIGURE 1 Disconnected electrical wires used in the sham acupuncture group.

SUPPLEMENTARY TABLE 1 Locations of Real and Sham Acupoints.

SUPPLEMENTARY TABLE 2 Data and Safety Monitoring Board Members.
