# Supplementary figures and images for: Efficacy of acupuncture in patients with mild Alzheimer’s disease and its impact on gut microbiota: Study protocol for a randomized sham-controlled trial
Source: Front Med (Lausanne). 2023 Feb 23;10:1014113. doi: 10.3389/fmed.2023.1014113 (PMC9996632; doi:10.3389/fmed.2023.1014113)

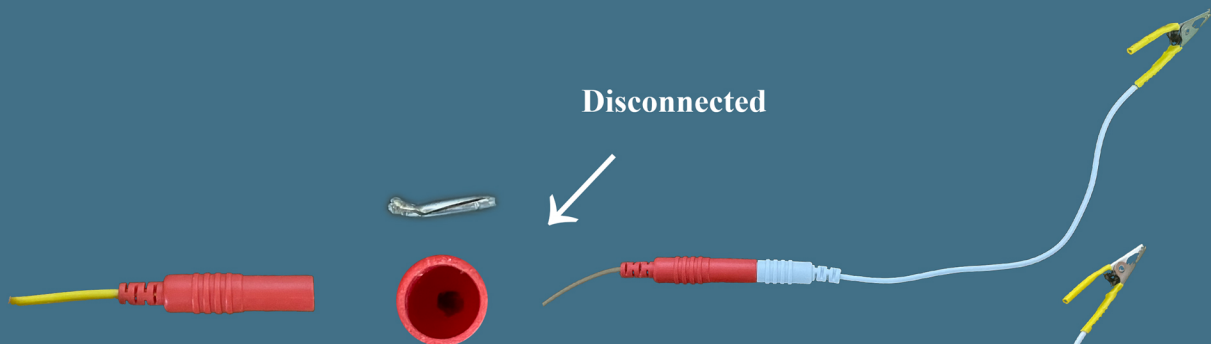

**Sham acupuncture group**

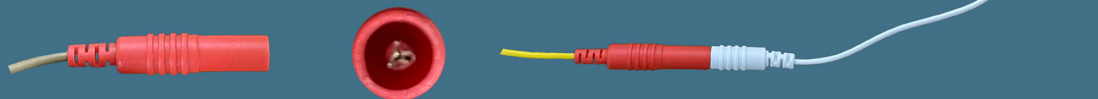

**Active acupuncture group**

Supplement: SUPPLEMENTARY FIGURE 1 — Disconnected electrical wires used in the sham acupuncture group. [file Image_1.PDF]
