# Supplementary material for: Efficacy of acupuncture in patients with mild Alzheimer’s disease and its impact on gut microbiota: Study protocol for a randomized sham-controlled trial
Source: Front Med (Lausanne). 2023 Feb 23;10:1014113. doi: 10.3389/fmed.2023.1014113 (PMC9996632; doi:10.3389/fmed.2023.1014113)
Supplement: SUPPLEMENTARY TABLE 1 — Locations of Real and Sham Acupoints. [file Table_1.PDF]

Supplementary Table 1. Locations of Real and Sham Acupoints.

Real/Sham Acupoint	Location	Direction of Insertion	Depth of Insertion (mm)
GV20	On the head, 5 cun superior to the anterior hairline, on the anterior median line	Oblique, 10-20°	8-15
EX-HN1	On the head, 1 cun anterior, posterior and lateral to GV20	Oblique, 10-20°	8-15
GV24	On the head, 0.5 cun superior to the anterior hairline, on the anterior median line	Oblique, 10-20°	5-8
PC6	On the anterior aspect of the forearm, between the tendons of tha palmaris longus and the flexor carpi radialis, 2 cun proximal to the palmar wrist crease	Perpendicular	8-15
HT7	On the anteromedial aspect of the wrist, radial to the flexor carpi ulnaris tendon, on the palmar wrist crease	Perpendicular	5-8
ST36	On the anterior aspect of the leg, on the line connecting ST35 with ST41, 3 cun inferior to ST35	Perpendicular	20-40
KI3	On the posteromedial aspect of the ankle, in the depression between the prominence of the medial malleolus and the calcaneal tendon	Perpendicular	8-15
SP6	On the tibial aspect of the leg, posterior to the medial border of the tibia, 3 cun superior to the prominence of the medial malleolus	Perpendicular	15-35
GB39	On the fibular aspect of the leg, anterior to the fibula, 3 cun proximal to the prominence of the lateral malleolus	Perpendicular	8-15
CV6	On the lower abdomen, 1.5 cun inferior to the centre of the umbilicus, on the anterior median line	Perpendicular	20-40
ST40	On the anterolateral aspect of the leg, lateral border of the tibialis anterior, 8 cun superior to the prominence of the lateral malleolus	Perpendicular	20-35
SP10	On the anteromedial aspect of the thigh, on the bulge of the vastus medialis muscle, 2 cun superior to the medial end of the base of the patella	Perpendicular	20-35
SA1	In the lumbar region, 5 cun lateral to GV5	Perpendicular	NA
SA2	In the lumbar region, 5 cun lateral to GV4	Perpendicular	NA
SA3	On the posterior aspect of the leg, 1 cun lateral to BL56	Perpendicular	NA

The locations of acupoints and the acupoints used for locating the sham acupoints are referenced to WHO Standard Acupuncture Point Locations[1]. A cun is a unit of measure that is used in traditional Chinese medicine to measure the length of a certain part of the body surface for the convenience of locating acupoints.

Reference

1. WHO Regional Office for the Western Pacific, WHO Standard Acupuncture Point

Locations in the Western Pacific Region, World Health Organization, Manila, Philippines, 2008.
