# Supplementary material for: Efficacy of acupuncture in patients with mild Alzheimer’s disease and its impact on gut microbiota: Study protocol for a randomized sham-controlled trial
Source: Front Med (Lausanne). 2023 Feb 23;10:1014113. doi: 10.3389/fmed.2023.1014113 (PMC9996632; doi:10.3389/fmed.2023.1014113)
Supplement: SUPPLEMENTARY TABLE 2 — Data and Safety Monitoring Board Members. [file Table_2.PDF]

Supplementary Table 2. Data and Safety Monitoring Board Members.

Name	Role	Affiliation	Responsibilities
Chunbo Li, MD	Chair	Shanghai Mental Health Center, Shanghai, China	Chair the DSMB discussion. Review the the protocol and progress of the trial with respect to ethical and safety standards, monitor the integrity of the data with respect to original study design, and provide advice on study conduct.
Shifen Xu, MD	Member	Shanghai Municipal Hospital of Traditional Chinese Medicine, Shanghai University of Traditional Chinese Medicine, Shanghai, China	Review the the protocol and progress of the trial with respect to ethical and safety standards, monitor the integrity of the data with respect to original study design, and provide advice on study conduct.
Ruiping Wang, MD	Member	Skin Disease Hospital of Tongji University, Shanghai, China	
